# Duration of Protection and Humoral Immune Response in Nile Tilapia (*Oreochromis niloticus* L.) Vaccinated against *Streptococcus agalactiae*

**DOI:** 10.3390/ani14121744

**Published:** 2024-06-09

**Authors:** Guilherme Alves de Queiróz, Tarcísio Martins França e Silva, Carlos Augusto Gomes Leal

**Affiliations:** Department of Preventive Veterinary Medicine, Veterinary School, Federal University of Minas Gerais, Belo Horizonte 31270-901, MG, Brazil; guiaqua7@yahoo.com.br (G.A.d.Q.); ailavet@yahoo.com.br (T.M.F.e.S.)

**Keywords:** antibody, ELISA, immune response, streptococcosis, vaccination

## Abstract

**Simple Summary:**

*S. agalactiae* is one of the main bacterial pathogens responsible for mortality outbreaks and economic losses in tilapia farming worldwide. Vaccination has proven to be the most efficient measure for the prevention and control of streptococcosis. Currently, several commercial vaccines against *S. agalactiae* are available for use in the tilapia industry. However, since tilapia has received the vaccine, very little attention has been paid to the duration of vaccine protection and the humoral immune response of fish post-vaccination. Our study provides valuable information regarding the long-term protection conferred by vaccination in Nile tilapia against infection caused by *S. agalactiae*, showing that vaccination with a single dose may protect tilapia against streptococcosis from 15 to 300 days post-vaccination. Based on the serological test results, the detection of anti-*S. agalactiae* IgM could be used as a non-lethal monitoring tool to assess the efficacy of vaccination programs until 180 dpv. However, vaccine protection over six months can be associated with other components of the fish immune system beyond the humoral immune response by IgM antibodies.

**Abstract:**

Streptococcosis caused by *Streptococcus agalactiae* (*S. agalactiae)* is a major bacterial disease affecting the production of Nile tilapia (*Oreochromis niloticus* L.), causing significant economic losses due to mortality in the growing phase. Vaccination is the most effective method for preventing streptococcosis on Nile tilapia farms. In Brazil, the major tilapia-producing regions have long production cycles (6–10 months) and harvest tilapias weighing over 900 g for fillet production. Thus, data on the duration of the humoral immune response and protection in farmed tilapia have not been reported or are poorly described. Furthermore, the efficiency of serological testing for the long-term monitoring of immune responses induced by vaccination against *S. agalactiae* has never been addressed. This study evaluated the duration of protection and humoral immune response induced in Nile tilapia vaccinated against *S. agalactiae* until 300 days post-vaccination (dpv). The immunization trial was composed of two groups: vaccinated (Vac), vaccinated intraperitoneally with a commercial vaccine, and unvaccinated (NonVac) group, injected fish with sterile saline solution. At 15, 30, 150, 180, 210, and 300 dpv, blood sampling was conducted to detect anti-*S. agalactiae* IgM antibodies using indirect Enzyme-Linked Immunosorbent Assay (ELISA), and the fish were challenged with pathogenic *S. agalactiae* to determine the duration of vaccine protection through relative percentage survival (RPS). Spearman’s rank correlation was performed between the ELISA optical density (OD) of vaccinated tilapia and the duration of vaccine protection (RPS). The mean cumulative mortality in NonVac and Vac groups ranged from 65 to 90% and less than 35%, respectively. The average RPS was 71, 93, 94, 70, 86, and 67% at 15, 30, 150, 180, 210, and 300 dpv, respectively. RPS revealed that the vaccine provided protection from 15 to 300 dpv. The specific anti-*S. agalactiae* IgM antibody levels were significantly higher in the Vac group than that non-Vac group up to 180 dpv. The vaccinated fish exhibited significant protection for up to 10 months after vaccination. There was a positive correlation between the antibody response and RPS. This study revealed that a single dose of commercial vaccine administered to Nile tilapia can confer long-term protection against *S. agalactiae* and that indirect ELISA can monitor the duration of the humoral immune response for up to six months following vaccination. Finally, vaccine protection over six months can be associated with other components of the fish immune system beyond the humoral immune response by IgM antibodies.

## 1. Introduction

Nile tilapia (*Oreochromis niloticus* L.) is one of the most important fish in aquaculture worldwide, being the main farmed species in Brazil, accounting for 60.8% (408.0 thousand tons) of national production [1,2]. Disease outbreaks are a major problem faced by farms because of the high levels of stress caused by high stocking density and poor water quality [3]. Streptococcosis outbreaks caused by *Streptococcus agalactiae* (*S. agalactiae*; Lancefield’s group B Streptococcus, GBS) are one of the main causes of economic losses in intensive Nile tilapia farms [3,4]. Antibiotic therapy administered orally through feed is a widely used measure for controlling streptococcosis in farms [5]. However, the continuous and indiscriminate use of antimicrobials on farms is a public health problem worldwide, mainly owing to drug resistance issues and safety concerns [6]. Vaccination is considered the most appropriate strategy for streptococcosis control in tilapia farms because it is associated with mortality prevention, improvement in survival rates and feed conversion ratio (FCR), and reduces antibiotic use [7,8].

Several commercial vaccines are available to prevent outbreaks caused by bacterial diseases in farmed fish worldwide, including streptococcosis in Nile tilapia. Generally, these vaccines comprise inactivated pathogen cells emulsified in oily adjuvants. The vaccines are administered intraperitoneally in a single dose (usually 0.05 mL per fish) and are recommended for fish with a body weight of at least 15 g [9].

The main tilapia-producing countries worldwide have a production cycle that takes four to seven months. In China, some of the largest tilapia-producing regions harvest fish with weights ranging from 300 to 500 g, reared in production cycles that take 5 to 7 months, whereas in Egypt, tilapia with final weights between 200 and 350 g are harvested during a similar period [10,11]. In Brazil, in the two regions responsible for the highest tilapia production in the country (South and Southeast), production cycles of 6–10 months are carried out to obtain tilapia with an average weight of 900 g or more [12]. The major reason for producing larger fish is associated with the industry demand for fillet production, which is the main tilapia product consumed in the Brazilian domestic fish market and a major export (chilled tilapia fillet) to the USA and other countries [12,13]. Because the production cycle is longer than six months, fish are raised over a longer period on farms and thus remain exposed to streptococcosis outbreaks during the growing phase. The main risk factors for *S. agalactiae* outbreaks in tilapia farms are high water temperature (>27 °C) and adult fish (fattening stage), being able to reach until 90% of mortality in affected batches [3,7]. Therefore, longer production cycles increase the risk of outbreaks in endemic farms or regions.

In this context, the duration of the humoral immune response and the protection conferred by vaccination against streptococcosis in farmed tilapia are important features for evaluating the efficiency of vaccination programs [14]. However, most commercial vaccines designed for tilapia to prevent *S. agalactiae* infections do not include these parameters in their package insert. Furthermore, the duration of the immune response in tilapia post-vaccination is not well established. Few studies have demonstrated the duration of the long-term humoral immune response in tilapia or other fish species vaccinated against *Streptococcus* spp. Fish vaccinated with a single intraperitoneal injection of vaccines composed of formalin-killed bacteria resulted in specific antibody production that was detectable up to 180 days post-vaccination (dpv) [15,16]. Thus, once the animals are vaccinated and maintained under cultivation for a long period (over six months), the duration of the protection and humoral immune response in fish until harvesting remains relatively unexplored.

More than 40% of Nile tilapia farmed in the country are vaccinated annually, and Brazil has among the highest vaccination rates for tilapia [17]. The evaluation of vaccine efficiency in vaccination programs is commonly performed by monitoring mortality rates and weight gain in vaccinated tilapia batches throughout the production cycle [18]. In addition, the vaccination rate is estimated by the macroscopic recognition of the deposit of the vaccine residue in the coelomic cavity of fish after the vaccination process [19]. However, vaccination by intraperitoneal injection does not guarantee that the fish will produce a protective immune response because this response can be affected by low water temperatures in atypical cold periods and under stressful conditions (e.g., hypoxia and intensive handling) in intensive production environments [3,20,21]. Therefore, laboratory tests are crucial for determining the profile of vaccine protection of immunized fish batches against infectious diseases at the farm level, similar to what occurs routinely in other segments of animal production, such as the broiler chicken and pork industries [22,23].

Serological methods have been used in terrestrial animals for purposes such as disease diagnosis, surveillance, and certification for movement and to demonstrate the efficacy of vaccination [24]. In finfish, serological methods are mainly used in research on immune responses resulting from the effects of diets, environmental parameters, pathogen diagnosis (e.g., disease surveillance), and evaluation of post-vaccination immune responses [25]. Enzyme-Linked Immunosorbent Assay (ELISA) is one of the most commonly used serological methods in finfish, mainly for the detection and measurement of immunoglobulin titers against bacteria during vaccine development, such as *S. agalactiae* in tilapia [26,27,28,29,30]. The main advantages of ELISA are the non-lethal nature and large-scale sampling of fish blood to obtain the serum required for testing [25].

This study aimed to evaluate the duration of protection and humoral immune responses induced in Nile tilapia vaccinated with *S. agalactiae* for 300 dpv. In addition, we assessed the efficiency of indirect ELISA as a tool for surveying and predicting the protective immune response induced by vaccination.

## 2. Materials and Methods

### 2.1. Ethical Consideration

This study was approved by the Ethics Committee for Animal Experiments of the Federal University of Minas Gerais, Brazil (CEUA-UFMG, protocol nº 235/2020).

### 2.2. Fish

The study was conducted in the Routine Bacteriology Laboratory and Aquatic Animal Diseases Laboratory at the Veterinary School of the Federal University of Minas Gerais, Minas Gerais, Brazil (EV/UFMG). A total of 250 male juveniles of Nile tilapia (*Oreochromis niloticus*, L.) weighing approximately 14.0 ± 4.00 g were purchased from a commercial hatchery. Prior to the experimental trial, ten fish were randomly selected and subjected to parasitological and bacteriological examinations to demonstrate freedom from subclinical infectious diseases, particularly by *S. agalactiae*.

Fish were acclimated in 310 L polyethylene circular tanks with aeration. The tanks were supplied with flow-through dechlorinated tap water (90 L/h) at a water temperature of 26 ± 1.0 °C for 60 days. They were subjected to an artificial photoperiod of 12L:12D (L: h of light, D: h of dark) and were fed to apparent satiation with Nutripiscis AL containing 36% crude protein (Presence, Brazil) twice a day.

### 2.3. Vaccination Trial

**Figure 1:**
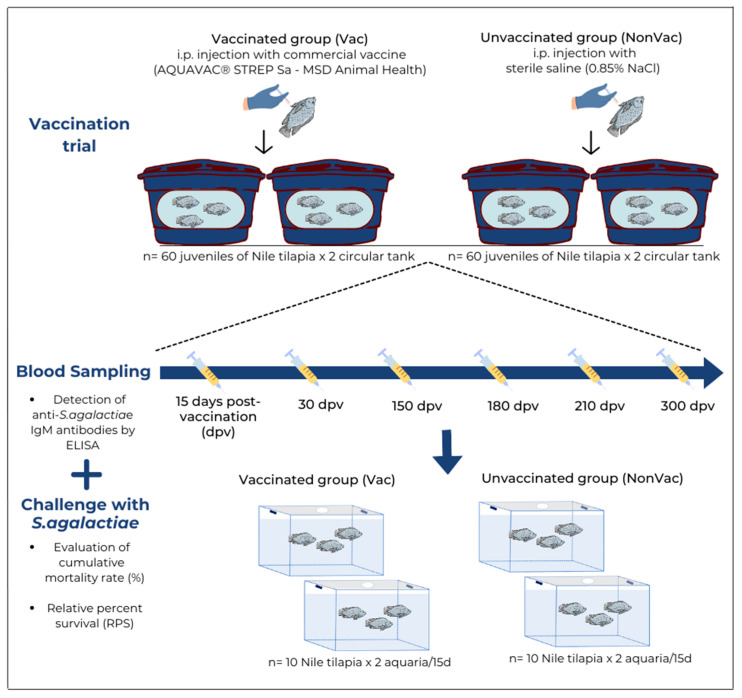
Schematic view of experimental design. Fish were intraperitoneally vaccinated with commercial vaccine against *S. agalactiae* (Vac group) and sterile saline (NonVac group). For both groups (n = 60 fish × 2 circular tank), six blood samples were taken at 15, 30, 150, 210, and 300 days post-vaccination (dpv) and were intraperitoneally challenged with a *S. agalactiae* strain (n = 10 fish × 2 aquaria). After the challenge, the fish were monitored daily for clinical signs and mortality for 15 days.

To evaluate the duration of protection and humoral immune responses induced by vaccination in Nile tilapia, an experimental vaccination followed by six bacterial challenges was performed over a period of 300 days. The experiment comprised two groups (vaccinated and unvaccinated). Each group had two replicates, as shown in Figure 1. Each experimental group consisted of 120 juvenile Nile tilapia (60 fish × 2 circular tanks) with an average weight of 68.13 ± 21.88 g. The vaccinated group (Vac) was vaccinated with commercial vaccine AQUAVAC^®^ STREP Sa (MSD Animal Health Batch number 006/19, Rahway, NJ, USA) containing 1.36 × 10^8^ colony-forming units (CFU)/mL of *S. agalactiae* serotype Ib, and unvaccinated group (NonVac) was injected with sterile saline (0.85% NaCl). Prior to vaccination, the fish were starved for 48 h and anesthetized through immersion in a benzocaine (Sigma-Aldrich, St. Louis, MO, USA) solution (100 mg/L) [31]. Vaccines were administered to tilapia via intraperitoneal injection (0.05 mL/per fish, manufacturer’s instructions), using 1 mL syringe and 26 G × ½″-0.45 × 13 mm needles. After injection, all fish were returned to their respective tanks.

The fish were monitored two times a day and the water temperature was maintained using a heater at 26 ± 2.0 °C. Fish were fed twice a day with a commercial feed (Nutripiscis TR, Presence, Brazil; 36% crude protein) at 3% of body weight throughout the experimental period. In addition, the following parameters were recorded: feeding behavior (anorexia, hyporexia, or normorexia), swimming behavior (apathy, lethargy, or erratic swimming), and clinical signs of the disease (darkness, ascites, corneal opacity, skin ulcers, and exophthalmia).

### 2.4. Blood Sampling

After primary immunization, six samplings were conducted at 15, 30, 150, 180, 210, and 300 dpv (Figure 1). In each blood sampling, 10 animals from each tank (10 fish × 2 tanks in the vaccinated group and 10 fish × 2 tanks in the unvaccinated group) were randomly selected and anesthetized as described above, and blood was collected via caudal venipuncture. The whole blood samples were maintained at room temperature for 1 h and then allowed to clot for 24 h at 4 °C. The serum was separated through centrifugation (4000× *g* at 4 °C for 10 min). The serum was stored at −80 °C for further evaluation using ELISA. Immediately after blood sampling, the fish were challenged with an intraperitoneal injection of a virulent strain of *S. agalactiae* as described below.

### 2.5. Experimental S. agalactiae Challenge

The pathogenic *S. agalactiae* strain SA583-19 was used to prepare bacterial suspensions. This strain was selected from a bacterial collection at the Routine Bacteriology Laboratory (EV/UFMG). Strain SA583-19 was isolated from the brain of diseased adult tilapia (average weight 129 g) obtained from an outbreak of meningoencephalitis on a Nile tilapia farm in Minas Gerais (Brazil) in 2019. This isolate was characterized in the laboratory as non-hemolytic and belonged to the capsular serotype Ib, following the method described in a previous study [32]. The median lethal dose 50% (LD_50_) of the isolate was determined in a preliminary experiment, which resulted in an LD50 of 2.81 × 10^3^ CFU/fish, calculated using the method described in a previous study [33].

For the challenge trial, the isolate was thawed, streaked onto 5% horse blood agar (5% HBA), and incubated at 28 °C for 48 h. Some colonies were selected, inoculated into Brain Heart Infusion broth (BHI; Oxoid, Hampshire, UK), and incubated at 28 °C for approximately 10 h with agitation (150 rpm). The optical density (OD) of the bacterial suspension was adjusted to an OD at 600 nm (OD_600_) of 0.033. Fish from the vaccinated and control groups were intraperitoneally (i.p.) injected with 0.1 mL of *S. agalactiae* contained an average of 5.21 × 10^5^ CFU per fish, as determined by direct plate counting [34]. Following inoculation, fish were placed in four 120 L aquariums according to their respective groups: Vac (10 fish × 2 aquaria) and NonVac (10 fish × 2 aquaria) (Figure 1). Fish were monitored twice a day, and the aquarium water temperature was maintained at 28.2 ± 0.55 °C. The following parameters were evaluated: feeding behavior, clinical signs of disease, and mortality. The experimental infection period was 15 d. During this period, the fish were fed commercial feed as mentioned above. Brain and kidney samples were aseptically collected from all animals that died during the experimental period or were still alive at the end of this period after euthanasia through benzocaine overdose (300 mg/L). The samples were inoculated onto 5% HBA and incubated at 28 °C for 48 h for bacterial re-isolation.

At the end of each challenge trial period, the RPS [35] was calculated using the end cumulative mortality as follows:

RPS (%) = 1 − (% Mortality of vaccinated fish/% Mortality of unvaccinated fish) × 100.

### 2.6. Evaluation of Humoral Immune Response by ELISA

An indirect ELISA was developed to detect the anti-*S. agalactiae* IgM antibodies in the serum of the fish at different time points after vaccination (15, 30, 150, 180, 210, and 300 dpv).

#### 2.6.1. Coating Antigen Preparation

*Streptococcus agalactiae* whole cells were inactivated according to the method described in previous study [36], with modifications. Briefly, SA583-19 isolate was cultured, as described in Section 2.5, to a concentration of 1.61 × 10^9^ CFU/mL. Bacterial cells were harvested through centrifugation at 11,000 rpm at 4 °C for 12 min. The supernatant was discarded, and the pellet was resuspended in phosphate-buffered saline (PBS) with a pH of 7.4 added with 1% formaldehyde (Dinâmica, Ariquemes, Brazil) and incubated at 4 °C under low agitation (50 rpm) for 24 h. Formalin-inactivated whole cells (or aqueous bacterin) were washed three times using centrifugation and resuspended in 0.05 M carbonate-bicarbonate buffer (Sigma-Aldrich cat. no. C3041, St. Louis, MO, USA) at a pH of 9.6. To confirm the inactivation of bacterial cells, an aliquot of aqueous bacterin was plated onto 5% HBA and incubated at 28 °C for 48 h to check for sterility. Subsequently, aqueous bacterin was adjusted to optical densities of 0.5, 1, and 1.5 at 600 nm, and then stored at −80 °C until use.

#### 2.6.2. Development of Indirect ELISA Assay

For indirect ELISA, all components of the test were chosen according to their minimal interference of non-specific binding (background) and the highest difference between the results for vaccinated and non-vaccinated fish. The indirect ELISA was standardized by block titration of different optical densities of aqueous bacterin (0.5, 1 and 1.5 at OD_600_), tilapia serum (1:20–1:200), and peroxidase-conjugated rabbit polyclonal anti-tilapia antibody (1:200–1:800). The aqueous bacterin 1.5 at OD_600_ (corresponding to 1.0 × 10^9^ CFU/mL per well) was superior to the others optical densities (0.5 and 1) as a coating antigen in terms of the significant difference between the serum assessed and lowered background. The optimal dilutions of the tilapia serum and rabbit polyclonal anti-tilapia antibodies were 1:40 and 1:400, respectively.

A high-binding ELISA 96-well microplate (Costar cat. no. 3590, Washington, DC, USA) was coated with 100 µL antigen (aqueous bacterin) and incubated in a moist chamber at 28 °C for 1 h. The plates were washed three times with PBS containing 0.05% Tween 20 (PBST; Amresco, Solon, OH, USA), and non-specific binding was blocked with 200 µL of blocking solution constituted by 5% powdered skim milk (Nestlé, São Paulo, Brazil) in PBS. After incubation for 2 h at 28 °C, the plates were washed five times with PBST. Ten tilapia serum samples were randomly selected from each group (five fish × two tanks of vaccinated and five fish × two tanks unvaccinated group) and were diluted in PBST with 1% powdered skim milk, and 100 µL of sample solution was added to each well. All serum samples were tested in triplicates. After incubation for 1 h at 28 °C, the plates were washed three times with PBST. Then, 100 µL of rabbit polyclonal anti-tilapia antibody peroxidase-conjugated (RheaBiotec, Campinas, Brazil) diluted in PBS with 1% powdered skim milk and 1% bovine serum albumin (Sigma-Aldrich, St. Louis, MO, USA) was added to each well and incubated for 15 min at 37 °C. The plates were washed three times with PBST and 100 µL 3,3′,5,5′-Tetramethylbenzidine substrate (TMB; Sigma-Aldrich, St. Louis, MO, USA) was added to each well. The ELISA reaction was stopped after 30 min at 28 °C through the addition of 50 µL of 1 M H_2_SO_4_. The absorbance of the plates was measured at 450 nm using a microplate reader (BioTek, Winooski, VA, USA). Antibody reactivity was reported as OD450 value. Internal *Streptococcus*-positive and *Streptococcus*-negative control samples were included in all assays to verify test reproducibility.

### 2.7. Statistical Analysis

All statistical analyses were performed using GraphPad Prism Software Version 5.01 (GraphPad Software Inc., San Diego, CA, USA). The mean OD_450_ values were obtained from the detection of anti-*S. agalactiae* antibodies through indirect ELISA and subjected to the Shapiro–Wilk test to assess normality. Subsequently, the mean OD_450_ values ± standard deviation (SD) results of the vaccinated group were compared to the control through a t-test at a 5% significance level. The mean cumulative mortality rates among groups were compared using Fisher’s exact probability test. Correlation analysis was performed between the mean OD450 values of the anti-S. agalactiae IgM antibody in the vaccinated group six times post-vaccination (15 to 300 dpv) and the RPS following the challenge trial using the Spearman method. *p* values lower than 0.05 were considered statistically significant.

## 3. Results

### 3.1. Duration of Vaccine Protection against S. agalactiae

To determine the duration of protection provided by commercial vaccines in tilapia, we performed challenge experiments at 15, 30, 150, 180, 210, and 300 dpv. No abnormal behavior or mortality was observed in the experimental groups after handling or vaccination. Overall, the challenge with *S. agalactiae* resulted in the first clinical signs of streptococcosis in diseased fish at 48 h post-infection. The animals displayed lethargy and anorexia and remained at the bottom of the aquarium. Four days post infection, anorexia, unilateral exophthalmia, corneal opacity, ascites, erratic swimming, and ulcers were observed at the base of the pectoral fin. In the six *S. agalactiae* experimental infections carried out, the cumulative mortality was calculated considering the period of 15 days after challenge (challenge period). The NonVac group experienced an elevated cumulative mortality rate (≥65%), whereas the Vac group showed a mortality rate of less than 35%, as shown in Table 1. For each challenge period, the cumulative mortality rates differed significantly between the control and vaccinated groups.

In terms of vaccine protection, the average RPS in Vac ranged from 67% to 94% (Table 1). In the first 15 dpv, this group had an average RPS value of 71%, demonstrating that the vaccine provided protection in tilapia against S. agalactiae infection from two weeks after primary immunization. A sharp increase in RPS was observed at 30 dpv (93%), followed by at 150 dpv (94%), which was the peak value (Table 1). A remarkable protection rate was observed in both periods, although there was an interval of 120 days between the challenge trial time points. Subsequently, slight variations in RPS were verified at 180 and 210 dpv, with values of 70% and 86%, respectively. At 300 dpv, the average RPS decreased to 67%. Despite this decrease, this result indicates that the commercial vaccine conferred protection to Nile tilapia, even after 300 dpv (i.e., 10 months).

In both groups, all moribund and dead fish were found to be positive for *S. agalactiae* infection by re-isolating bacteria on 5% HBA from brain and kidney. All vaccinated fish that survived up to 210 dpv and were euthanized by the end of the challenge trial showed negative bacteriological results. However, at 300 dpv, one surviving fish in Vac (1/14) was positive for *S. agalactiae* infection at bacteriology, suggesting the presence of a carrier animal.

### 3.2. Detection of Antibody Response

Tilapia blood in the vaccinated and control groups was collected at 15, 30, 150, 180, 210, and 300 dpv, and anti-*S. agalactiae* IgM antibody was successfully detected using indirect ELISA.

**Figure 2:**
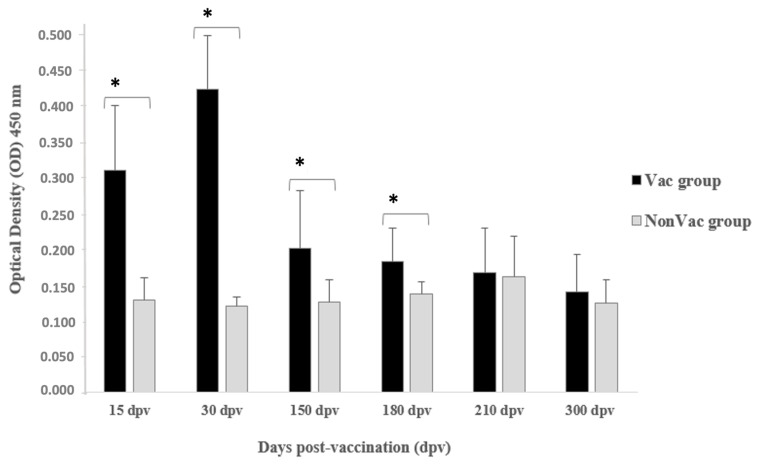
Specific anti-*S. agalactiae* IgM levels (ELISA optical density, OD) in Nile tilapia (*Oreochromis niloticus* L.) NonVac group (grey) and Vac group (black) with commercial vaccine pre-challenge with *S. agalactiae* at 15, 30, 150, 180, and 300 dpv. Each point represents mean value ± SD (n = 10 per group). The *t*-test comparison of the unvaccinated and vaccinated groups is shown. * represents a significant difference from unvaccinated group (*p* < 0.05).

In NonVac, there was no detection of anti-*S. agalactiae* IgM antibodies in the Nile tilapia serum over the entire experimental period, as shown in Figure 2. The mean OD_450_ values before the challenge ranged from 0.120 ± 0.013 to 0.162 ± 0.056 (Table 2). By contrast, the vaccine-induced relatively high antibody levels in Nile tilapia vaccinated with anti-*S. agalactiae* IgM antibodies were detected in almost all sampling periods. This group showed OD_450_ readings ranging from 0.140 ± 0.052 to 0.423 ± 0.075 (Table 2; Figure 2). Significant specific antibody levels were detected after the second week post-vaccination when compared to the NonVac group (*p* < 0.05) and reached the highest mean OD_450_ value at 30 dpv (0.423 ± 0.075).

Anti-*S. agalactiae* IgM antibody levels began to drop in fifth month post-vaccination and continued to decrease regularly and steadily until the end of the study. At 150 and 180 dpv, the mean OD_450_ was 0.183 ± 0.047 and 0.167 ± 0.062, respectively. Nevertheless, the mean OD_450_ value of the Vac group was significantly different (*p* = 0.0119) from that of the unvaccinated fish up to 180 dpv. In the last two periods evaluated, 210 and 300 dpv, the mean OD_450_ values were not significantly different (*p* > 0.05) from those measured for the control group.

To investigate the correlation between the anti-*S. agalactiae* IgM antibody levels detected by indirect ELISA in the serum of vaccinated Nile tilapia and the duration of vaccine protection (RPS) achieved at the end of each challenge period, a Spearman analysis was performed. There was a positive correlation between antibody response and RPS; however, this was not statistically significant (r = 0.714, *p* = 0.068).

## 4. Discussion

Based on the vaccination trial conducted in this study, the commercial vaccine conferred significant protection to Nile tilapia after the challenge with *S. agalactiae* over the experimental period. Interestingly, the RPS values obtained after the challenge trial were statistically significant at 15 dpv, at 71%. That early protection can also be associated with strong innate immune response induced by the vaccine [37]. Previous studies demonstrated that inactivated *S. agalactiae* whole-cell vaccine administered intraperitoneally provides a generally higher protective effect (RPS > 60%) in experimentally challenged Nile tilapia [8,14]. However, most studies performed challenge trials from 28 to 30 dpv; therefore, data regarding vaccine protection in tilapia at two weeks post-vaccination remain unavailable. However, at 15 dpv, Chen et al. [38] reported average RPS values of 42, 86, and 100% in Nile tilapia vaccinated (separately) with ten formalin-killed *S. agalactiae* vaccines prepared using distinct PFGE genotypes, followed by a challenge with homologous or heterologous bacterial strains with the identical genotypes. Conversely, Li et al. [39] generated a live-attenuated *S. agalactiae* vaccine for Nile tilapia via continuous passage in vitro and evaluated its protective efficacy against experimental *S. agalactiae* infection 15 and 30 days after different routes of immunization (via injection, immersion, and oral administration). Tilapia vaccinated and challenged at 15 dpv (i.p. injection) had an elevated protection level, with an average RPS of 96.88%. However, both studies used *S. agalactiae* strains belonging to serotype Ia, and the latter described another designed vaccine (live attenuated) for Nile tilapia, which was quite different from that used in the present study. Therefore, our results of vaccine protection found at 15 dpv (average RPS value of 71%) provide unprecedented data regarding vaccine protection level based on inactivated *S. agalactiae* whole-cell vaccine for Nile tilapia.

In Brazil, there are no studies describing the duration of vaccine protection in Nile tilapia vaccinated with commercial vaccines, although technical brochures of some vaccines marketed nationally (e.g., the vaccine used in this study) have reported protection around seven months post-vaccination [18]. However, our results showed that the vaccination with a single dose conferred protection for Nile tilapia against *S. agalactiae* challenge, with RPS values above 60% up to 300 dpv (10 months). This outcome is superior to that reported by the vaccine manufacturer and a previous study by Pasnik et al. [16]. The authors evaluated the protection provided by a vaccine developed using the extracellular product fraction and formalin-killed whole cells of *S. agalactiae* at 47, 90, and 180 dpv. Based on the RPS calculations inferred from this study, the vaccine promoted RPS by 61%, 45%, and 47% at 47, 90, and 180 dpv, respectively. These data must be interpreted with caution because the low RPS values generated by the vaccine may be related to the absence of an oil-based adjuvant in the vaccine formulation, as its inclusion in the immunobiological product generally improves survival and promotes long-lasting protection [40,41]. Given the average RPS value (67%) observed at 300 dpv in the present study, this period could be considered a baseline for long-lasting vaccine protection in vaccinated tilapia, as no other study has evaluated the protection rate achieved after vaccination beyond 180 days [16]. This finding highlights the importance of long-term vaccine protection in intensive tilapia farms, especially in countries that produce larger fish (≥900 g) for fillet production and possess longer production cycles (over six months), such as Brazil. In addition, tilapia vaccinated with a single dose may display prolonged vaccine protection (up to 10 months) against mortality caused by *S. agalactiae*, which is frequently reported at farms in the growing phase [4,12].

ELISA is used to assess the vaccine-induced humoral immune response during the development of vaccines against bacterial diseases in fish species that are globally important for aquaculture [25]. In studies on vaccines against *S. agalactiae* in tilapia, ELISA was shown to be adequate for detecting and measuring anti-*S. agalactiae* in the sera of vaccinated fish [26,27,28,29,30]. Normally, ELISA is used to evaluate the humoral immune response in fish between 28 and 30 days after vaccination, which makes it impossible to determine the long-term duration of this response. To the best of our knowledge, the current study is the first to use indirect ELISA to detect anti-*S. agalactiae* IgM antibodies in Nile tilapia within 300 days of post-vaccination.

Furthermore, an important outcome of fish vaccination is the correlation between the protective effect and specific antibody response generated by the vaccine [42]. This approach has been reported as an alternative to the traditional in vivo immunization challenge batch potency testing of fish vaccines [37] and for evaluating the efficacy of vaccination [16]. For these reasons, some studies have employed ELISA to measure specific antibody levels (e.g., OD value) post-vaccination, followed by the determination of RPS values and survival rates after fish are challenged with distinct bacterial pathogens. Bricknell et al. [43] investigated the presence of protective antibodies and their correlation with protection in Atlantic salmon (*Salmo salar*) that were intraperitoneally administered an inactivated *Aeromonas salmonicida* vaccine containing iron-regulated outer-membrane proteins and secretory polysaccharides. At 56 and 240 dpv, the fish were challenged experimentally with the virulent *A. salmonicida* strain. The authors reported a strong correlation between the resistance of vaccinated fish following challenges and antibody response. Villumsen et al. [44] found a significant positive correlation between increasing levels of specific antibodies in rainbow trout (*Oncorhynchus mykiss*) serum vaccinated with commercial and experimental vaccines against *A. salmonicida* and increased survival after challenge with this bacterium at 18 weeks post-vaccination. Similarly, Raida et al. [45] observed an association between an increase in IgM antibody titers in rainbow trout immunized against *Yersinia ruckeri* and significantly reduced mortality during a bacterial challenge. In Nile tilapia, sporadic studies have reported a correlation between antibody responses in fish immunized against *S. agalactiae* and protection following a bacterial challenge. For example, Pasnik et al. [16,46] observed that increased pre-challenge antibody OD levels in actively and passively vaccinated tilapia were significantly correlated with post-challenge survival in *S. agalactiae*.

The standardized indirect ELISA in the present study satisfactorily detected the presence of anti-S. agalactiae IgM antibodies in the serum of vaccinated fish (Table 2) allowed us to evaluate the correlation between the tilapia antibody response and the duration of vaccine protection post-challenge with *S. agalactiae*. There was a positive correlation between antibody response and RPS (r = 0.714), although this result was not statistically significant (*p* = 0.068). This outcome was similar to that observed by Romstad et al. [37], who found a correlation (r = 0.75; *p* < 0.059) between antibody responses in Atlantic salmon vaccinated with commercial vaccines against *A. salmonicida*, and protection in fish challenged with this pathogen. However, our results supported previous findings that humoral immune responses in vaccinated Nile tilapia are associated with protection against *S. agalactiae* challenge [46]. The present study provides tentative initial evidence that indirect ELISA can serve as an alternative tool for monitoring vaccination efficiency and the protective immune response generated by vaccination in intensive tilapia cultures, since the current strategies are usually based on the evaluation of the vaccinator’s performance post-vaccination, improvement of weight gain, and monitoring mortality rates of the vaccinated tilapia during the production cycle [18,19].

Several studies have demonstrated the importance of immunoglobulins (or antibodies) in teleost fish in protecting against several infectious agents [47,48,49]. Teleosts possess three classes of effector immunoglobulins: IgM, IgD, and IgT/Z. IgM and IgD are found in most species of fish, while IgT/Z is the most recently described immunoglobulin and is present in only a few species of fish [50]. IgM is the most abundant immunoglobulin isotype in teleost fish and acts as the principal mediator of the humoral immune response at the systemic level and in some mucosal compartments [51]. Nile tilapia possess three immunoglobulin isotypes (IgM, IgD, and IgT), with IgM described as the main immunoglobulin isotype responsible for promoting protection against bacterial infections in tilapia [48,52,53]. In the present study, significant anti-*S. agalactiae* IgM levels were detected in the vaccinated group from the first 15 dpv compared to the control group (0.311 ± 0.090 vs. 0.129 ± 0.031, *p* < 0.05). The highest IgM antibody level was detected at 30 dpv (0.423 ± 0.075, *p* < 0.05) followed by decreases at 150 and 180 dpv. Regardless of this decrease in IgM antibody levels, a significant difference was observed between anti-*S. agalactiae* IgM antibody levels in the sera of vaccinated and unvaccinated tilapia up to 180 dpv (*p* = 0.0119). This result is in agreement with the findings of Pasnik et al. [16], who showed that tilapia vaccinated with formalin-killed whole cells of *S. agalactiae* formulated with extracellular product exhibited significant means of specific anti-*S. agalactiae* antibody up to 180 dpv. In addition, no significant differences were found in anti-S. agalactiae IgM levels in vaccinated fish when compared to the control group at 300 dpv, although the vaccine promoted an RPS of 67%, which represents the minimum RPS suggested for vaccination in fish (higher than or equal to 60%) [54]. Based on these results, we hypothesized that, in addition to the production of anti-*S. agalactiae* IgM antibodies, the vaccine may elicit a response of another isotype-specific immunoglobulin that contributes to the prolonged protection of vaccinated Nile tilapia against *S. agalactiae*. A previous study indicated that Nile tilapia significantly expressed the mRNA of specific IgD immunoglobulins against *S. agalactiae* following an intraperitoneal challenge with a pathogenic isolate of this bacterium [53]. Furthermore, Mai et al. [55] reported a significant increase in IgD mRNA expression in tilapia that were experimentally vaccinated with heat- and formalin-killed vaccines against *Tilapia tilapinevirus*. Therefore, the long-term protection observed in vaccinated Nile tilapia in the present study could be effectively associated with the presence of IgD during the immune response against *S. agalactiae* infection. Further investigations are required to confirm and validate this hypothesis.

## 5. Conclusions

The results of this investigation revealed that a single dose of a commercial vaccine administered to Nile tilapia promoted protection for up to 10 months after vaccination. There was a positive correlation between anti-*S. agalactiae* IgM antibodies in vaccinated tilapia and protection following *S. agalactiae* challenge. Indirect ELISA showed the potential for monitoring the efficiency of the vaccination process. This test can be used to detect the anti-*S. agalactiae* IgM antibodies up to six months following vaccination but is not a suitable predictor of the duration of the humoral immune response for periods longer than 180 days. Finally, the vaccine protection conferred by Nile tilapia vaccinated against S. agalactiae at 180 dpv was associated with other components of the fish immune system beyond the humoral immune response of IgM antibodies.

## Figures and Tables

**Table 1:** Cumulative mortality rate and relative percentage of survival (RPS) of Nile tilapia (*Oreochromis niloticus* L.) non-vaccinated and vaccinated with commercial vaccine, following challenges with S. agalactiae at 15, 30, 150 180, and 300 days post-vaccination (dpv). Different superscript letters indicate significant differences (*p* < 0.05) between groups within each sampling dpv.

Experimental Group	Days Post-Vaccination (dpv)	No. Fish Challenged	Cumulative Mortality (%)	RPS (%)	*p*-Value *
NonVac	15	20	85 ^a^	71	0.0003
Vac	15	20	25 ^b^		
NonVac	30	20	70 ^a^	93	˂0.0001
Vac	30	20	5 ^b^		
NonVac	150	20	80 ^a^	94	˂0.0001
Vac	150	20	5 ^b^		
NonVac	180	20	65 ^a^	70	0.0095
Vac	180	20	20 ^b^		
NonVac	210	20	70 ^a^	86	0.0002
Vac	210	20	10 ^b^		
NonVac	300	20	90 ^a^	67	0.0002
Vac	300	20	30 ^b^		

* p value: Fisher’s exact test.

**Table 2:** ELISA detection of anti-*S. agalactiae* IgM antibodies of Nile tilapia (*Oreochromis niloticus* L.) non-vaccinated and vaccinated with commercial vaccine pre-challenged with *S. agalactiae* at 15, 30, 150 180, and 300 dpv. Data are reported as mean ± SD (n = 10).

Experimental Group	Days Post-Vaccination (dpv)	OD_450_ Pre-Challenge (Mean ± SD)	IC95% *	*p*-Value **
NonVac	15	0.129 ± 0.031	0.106–0.151	<0.0001
Vac	15	0.311 ± 0.090	0.246–0.375	
NonVac	30	0.120 ± 0.013	0.110–0.129	<0.0001
Vac	30	0.423 ± 0.075	0.369–0.477	
NonVac	150	0.126 ± 0.031	0.103–0.149	0.0142
Vac	150	0.201 ± 0.081	0.143–0.260	
NonVac	180	0.138 ± 0.017	0.125–0.150	0.0119
Vac	180	0.183 ± 0.047	0.149–0.217	
NonVac	210	0.162 ± 0.056	0.122–0.202	0.8501
Vac	210	0.167 ± 0.062	0.122–0.212	
NonVac	300	0.124 ± 0.033	0.100–0.148	0.4223
Vac	300	0.140 ± 0.052	0.103–0.177	

* IC 95%: confidence interval of 95%. ** *p* value: *t*-test comparison (*p* < 0.05).

## Data Availability

Data contained within the article.
